# Thirty Days without a Bite: Wernicke’s Encephalopathy in a Patient with Paranoid Schizophrenia

**DOI:** 10.4172/2329-6895.1000182

**Published:** 2014-09-25

**Authors:** Mélanie Langlois, Marie-Claire Doré, Robert Laforce

**Affiliations:** 1 Department of Neurological Sciences, University Hospital of Quebec, Canada; 2 Faculté of Medicine, Laval University, Quebec, Canada; 3 Clinique Interdisciplinary Memory (CIME), University Hospital of Quebec, Canada

**Keywords:** Wernicke’s Encephalopathy, Schizophrenia, Vitamin deficiency, Cognition, Neuroimaging, Wernicke-Korsakoff syndrome

## Abstract

Wernicke’s Encephalopathy (WE) is a preventable neurologic condition characterized by altered mental status, ophthalmoplegia, and ataxia. Although historically associated with alcoholism, a few authors have described WE in patients with non-alcohol related psychiatric disorders. We report herein the case of a 36-year-old young man with paranoid schizophrenia who was brought to hospital for confusion and difficulties with his vision. His roommate said he had gone about thirty days without eating ‘…because he was on a slimming cure’. History and physical examination suggested WE as a result of isolation and poor diet leading to nutritional deficiency. This was confirmed by brain magnetic resonance imaging showing classic thalamic, mammillary bodies and brainstem lesions. Of note, his cognitive profile was far more heterogeneous than what had classically been described in the literature and involved both cortical and subcortical pathology, generating memory but also significant executive deficits. Intravenous treatment with thiamine was given and our patient showed mild improvements in visual acuity and nystagmus. However, persistent cognitive and physical disabilities consistent with Korsakoff syndrome remained, and he now lives in a supervised home. This case illustrates the tragic consequences of nutritional deficiencies in a patient with paranoid schizophrenia. The threshold to suspect WE in schizophrenic patients should be lowered and in doubt prophylactic parenteral thiamine should be administered.

## Introduction

Wernicke’s Encephalopathy (WE) presents with a classic triad of acute neurological symptoms including confusion, ophthalmoplegia, and ataxia secondary to thiamine (vitamin B1) depletion [1]. Only a third of patients show the complete triad and therefore, WE can be a challenging diagnosis [2]. In approximately 85% of patients with WE, persistent neurobehavioral changes occur and are labeled Korsakoff syndrome (KS) (see [3], for a review). A great majority of patients with acute WE and/or chronic KS (the combined disorder is termed Wernicke-Korsakoff syndrome [WKS]) are reportedly never recognized. Although WKS most often affects people with a nutritional deficiency related to alcoholism, the syndrome can result from inadequate supply of thiamine of any cause (e.g., gastric bypass surgery, immunodeficiency syndromes, malignancies, hyperemesis gravidarum, liver disease, hyperthyroidism, or severe anorexia). Generally under-recognized is WKS in patients with psychiatric disorders who do not abuse alcohol or suffer from anorexia [4]. We report herein the case of a 36-year-old young man with paranoid schizophrenia who was brought to hospital for confusion and difficulties with his vision. According to history, this individual had gone thirty days without eating any food.

## Case Report

A 36-year-old man (YB) was brought to hospital for confusion and difficulties with his vision. Past medical history revealed that he had first presented with psychotic symptoms at the age of 24 in the context of NMDA intoxication. He was first treated for psychotic symptoms with risperidone at 28 following a series of emergency room consultations. A diagnosis of paranoid schizophrenia was made at the age of 33. Follow-up notes suggested poor compliance with his medications (Quetiapine 200 mg hs) but an overall stable psychiatric disease.

Upon admission, it appeared that YB’s landlord had recently reported to our patient’s sister that when he came to collect the rent, YB had lost ‘a lot of weight, maybe 50 pounds!’ His roommate said YB had gone about thirty days without eating ‘…because he was on a slimming cure’. He denied drinking alcohol or using drugs. Medications had not changed.

Examination revealed disorientation, dehydration, xeroderma and poor hygiene. Mental exam revealed an immature and passive patient, who provided the medical team with conflicting answers. There were no active psychotic elements. Blood pressure was 167/109. Tachycardia was only noted during transportation to hospital, and other vitals were normal. Neurological exam revealed a large amplitude upbeat nystagmus, maximal in upward gaze, with slight bilateral 6th nerve palsy (see Video), optic disc pallor OD, and significant peripapillary hemorrhage OS. Visual acuity was significantly decreased. There were no signs of weakness and reflexes were intact. He showed ataxia and mild dysmetria. YB was unable to recognize familiar faces (his sister, for example) and he showed retrograde and anterograde amnesia.

**Figure 1:**
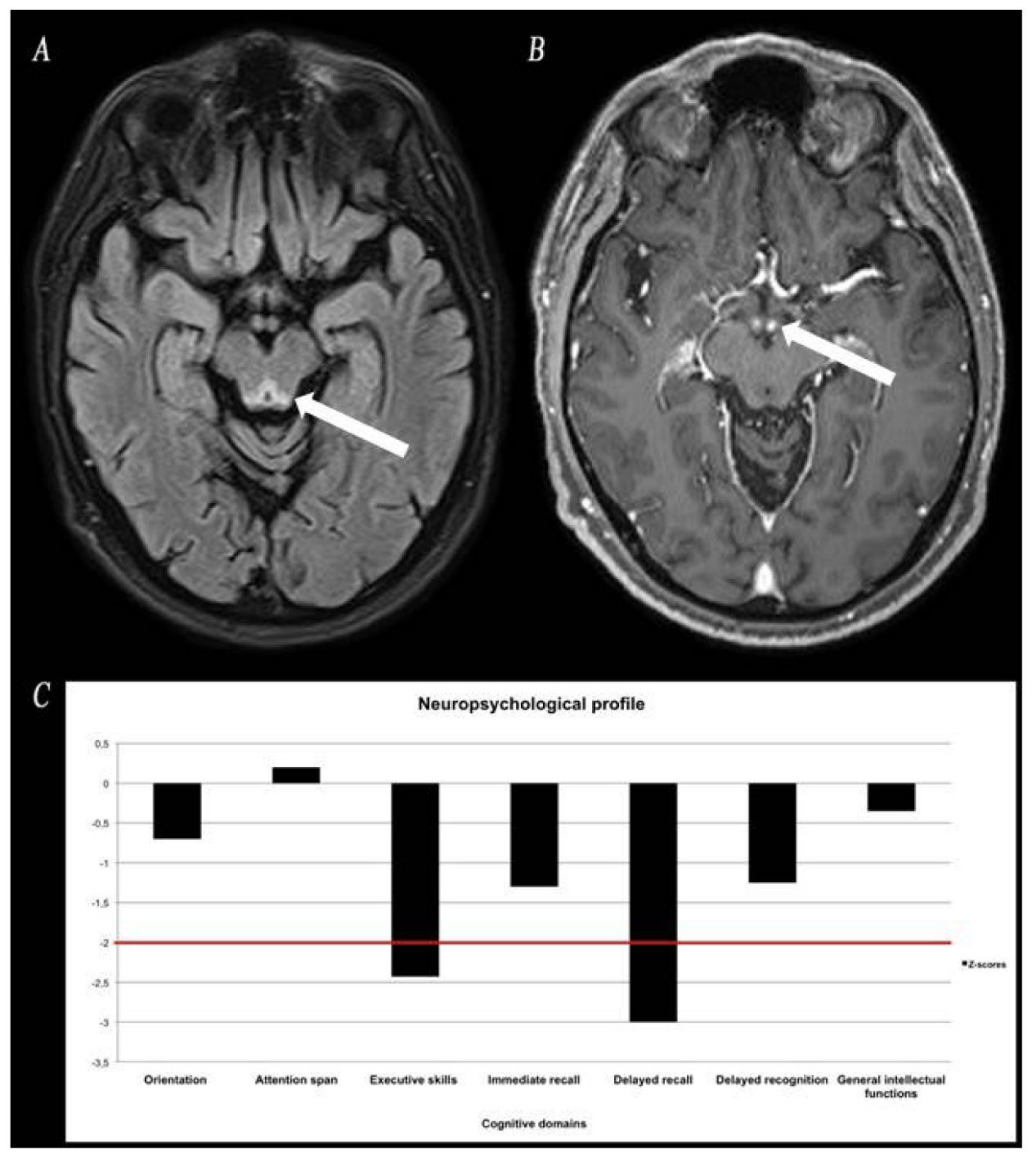
A. MRI of the brain showing increased T2/FLAIR in periaqueductal area. B. A 2nd MRI performed seven days after initial study revealed bilateral enhancement of the mammillary bodies on T1/gadolinium sequence. C. Neuropsychological assessment conducted two weeks after admission documented significant deficits in memory retrieval processes and executive skills.

**Figure 2:**
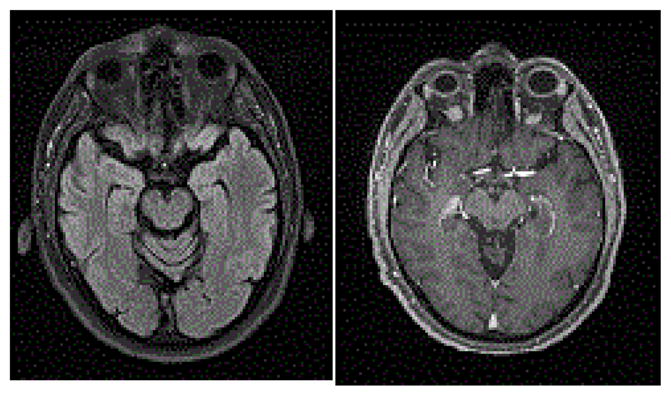
A. Resolution of periaqueductal hyperintensities, and B. mammillary bodies’ enhancement on a 3rd MRI conducted five weeks after admission. These figures document complete resolution of the abnormalities described on Figure 1.

He was admitted to Neurology and an extensive investigation was launched. Results showed increased hemoglobin, slightly elevated blood glucose and hepatic enzymes, with borderline pre-albumin (180 g/L; range 180–370 g/L) and normal albumin. Ketonuria, a simple marker of starvation, was not documented. Iron was slightly decreased but ferritin was normal. Cardiac, renal, thyroid, B12/folate, autoimmune/inflammatory tests, HIV, anti-GQ1b, and paraneoplastic panel (plus whole-body PET scan) were unremarkable. CSF showed slightly elevated glucose (5.8 mmol/L) and proteins (0.69 g/L) while VDRL and PCR herpes/tuberculosis/mycobacteria were negative. Vitamin B1 was only measured after heavy replenishment (i.e., thiamine 500 mg IV tid for five days), unfortunately, and therefore was normal. EMG/nerve conduction studies were intact. MRI of the brain revealed increased T2/FLAIR in periaqueductal area (Figure 1a). A 2nd MRI performed seven days after initial study revealed near resolution of periaqueductal hyperintensities but bilateral enhancement of the mammillary bodies (Figure 1b), which completely vanished on a 3rd MRI conducted five weeks after admission (Figure 2).

Neuropsychological assessment (Figure 1c) conducted two weeks after admission revealed intact orientation, attention span, and general intellectual functions. Significant deficits were found on executive tests (initiation, cognitive flexibility, working memory). Unexpectedly, YB’s memory profile did not show the classic anterograde amnesia characterized by encoding and consolidation dysfunctions. Rather, a frontal-subcortical pattern of memory deficits was seen on delayed measures where free recall was impaired but recognition was preserved. There was no confabulation. Behaviorally, he was apathetic, immature and irritable.

## Discussion

We report herein the case of a 36-year-old young man with paranoid schizophrenia who developed WE likely as a result of nutritional deficiencies. Because YB’s initial presentation was so intriguing, simple markers of starvation such as ketonuria or physical indicators of poor nutrition were not fully documented. Serial brain MRIs confirmed the location and reversible nature of lesions classically associated with WE. Despite early treatment with thiamine, our patient was left with permanent neurological and cognitive disabilities consistent with KS. Our Psychiatry team could not identify any worsening in his schizophrenic symptoms either before, during or after the events but one cannot help but think that his disease likely played a role in the initial thought of embarking on a strict slimming cure.

Only a few authors have described cases along the WKS continuum in patients with nutrional deficiencies not attributable to alcoholism or anorexia (see [4], for a review). Our patient was not in psychosis at the time of presentation but we hypothesized that the negative symptoms of schizophrenia might have caused his behavior of isolation, social withdrawal and poor diet leading to nutritional deficiency. Epidemiological studies suggest that individuals with WE typically have a younger age of onset than those with other forms of dementia, are more likely to be male, and often are socially isolated [3]. The cognitive profile is more heterogeneous than originally documented and involves both cortical and subcortical pathology. More specifically, memory retrieval and executive (planning, organizational) deficits are increasingly recognized [5,6].

Follow-up visits with YB revealed mild improvements in visual acuity, upbeat nystagmus, and the peripapillary hemorrhage OS had resolved. Unfortunately, significant cognitive (memory, executive) and behavioral deficits persisted on repeat neuropsychological examination seven months after discharge, and he now lives in a supervised home. This is observed despite near complete resolution of the lesions on brain MRI. Obviously we will never be able to fully ascertain whether an earlier intervention would have changed the course of this young man’s neurological presentation. Considering that schizophrenic patients are at considerable risk of malnutrition irrelative to alcohol consumption, the threshold to suspect WE in these patients should be lowered and in doubt prophylactically parenteral thiamine should be administered without delay.

## Supplementary Material

Video

## References

[1] Sechi G, Serra A (2007). Wernicke’s encephalopathy: new clinical settings and recent advances in diagnosis and management. Lancet Neurol.

[2] Cerejo R, Newey C, Stillman M (2013). Wernicke encephalopathy: diagnostically deceptive but treatable. Neurology.

[3] Ridley NJ, Draper B, Withall A (2013). Alcohol-related dementia: an update of the evidence. Alzheimer’s Research and Therapy.

[4] McCormick LM, Buchanan JR, Onwuameze OE, Pierson RK, Paradiso S (2011). Beyond alcoholism: Wernicke-Korsakoff syndrome in patients with psychiatric disorders. Cogn Behav Neurol.

[5] Kessels RP, Kortrijk HE, Wester AJ, Nys GM (2008). Confabulation behavior and false memories in Korsakoff’s syndrome: role of source memory and executive functioning. Psychiatry ClinNeurosci.

[6] Oscar-Berman M (2012). Function and dysfunction of prefrontal brain circuitry in alcoholic Korsakoff’s syndrome. Neuropsychol Rev.

